# Ataxia-Telangiectasia in Resource-Limited Settings: A Diagnostic Challenge

**DOI:** 10.7759/cureus.92265

**Published:** 2025-09-14

**Authors:** Ebenezer Adeyemi, Racheal Idowu, Adewale Adefolaji, Folasade Adeyemi, Paul Olowoyo

**Affiliations:** 1 Pediatrics, Federal Teaching Hospital, Ido Ekiti, NGA; 2 Pediatrics, Afe Babalola University, Ido Ekiti, NGA; 3 Radiology, Obafemi Awolowo University Teaching Complex, Ife, NGA; 4 Internal Medicine, Federal Teaching Hospital, Ido Ekiti, NGA

**Keywords:** alpha-fetoprotein, ataxia-telangiectasia, atm gene, cerebellar atrophy, immunodeficiency

## Abstract

Ataxia-telangiectasia (A-T) is a rare autosomal recessive disorder characterized by cerebellar ataxia and oculocutaneous telangiectasias, with increased malignancy risk from radiation-induced DNA double-strand breaks. Its multisystem involvement makes diagnosis challenging, and confirmation requires identifying biallelic pathogenic variants in the ATM gene. We report an eight-year-old boy with unsteady gait and bilateral ocular telangiectasia. He had no recurrent infections, and immunoglobulin (Ig) levels were normal, except for low IgA. Although serum alpha-fetoprotein was elevated and MRI demonstrated cerebellar atrophy, findings suggestive of A-T, definitive genetic testing for ATM mutations was not available

## Introduction

Ataxia telangiectasia (A-T) is a rare autosomal recessive disease first described in 1926 by Syllaba and Herner [1]. It is a multisystemic disorder characterized by ataxia, oculocutaneous telangiectasia, and immunodeficiency affecting both cellular and humoral immunity. A-T predisposes individuals to cancer due to DNA breakage caused by radiation. Ataxia is often the earliest manifestation, followed by the ocular telangiectasia [2].

Other clinical features may include nystagmus, mild-to-moderate cognitive impairment, tremors, dystonia, cutaneous telangiectasias, recurrent sinopulmonary infections, and an increased risk of malignancy [3]. Laboratory abnormalities often comprise elevated alpha-fetoprotein, lymphopenia, and immunoglobulin deficiencies. Magnetic resonance imaging (MRI) typically reveals cerebellar atrophy.

Management is supportive. The prognosis is poor, as ataxia is usually progressive, with loss of independent ambulation occurring by 10-12 years of age (though this may occur earlier or later). Most individuals with classic A-T eventually require a wheelchair for mobility. Median survival is approximately 17-25 years [4].

We report this rare case of A-T in an eight-year-old boy who presented with abnormal gait and ocular telangiectasia. The case merits publication because it illustrates the diagnostic challenges clinicians face in resource-poor settings, where reliance on clinical features, biochemical markers, and neuroimaging findings often substitutes for confirmatory molecular testing. Furthermore, the case contributes to the limited body of literature on A-T from this region, highlighting the importance of clinical vigilance in recognizing rare neurogenetic disorders. Sharing this experience will not only raise awareness but also encourage improved diagnostic capacity and early intervention strategies for affected patients in similar environments.

## Case presentation

An eight-year-old boy presented with an unsteady gait and bilateral conjunctival telangiectasia. The gait abnormality was first noted at 15 months of age and had progressively worsened over time. Prenatal, perinatal, and postnatal histories were unremarkable. There was no history of fever, seizures, or trauma. Bilateral eye redness was observed from the age of two years, with gradual progression, but without associated discharge, itching, or blurred vision. Developmental milestones were comparable to his older siblings until the onset of gait disturbance at one year. There was no family history of similar illness or any known genetic disorders on either the paternal or maternal side.

**Figure 1:**
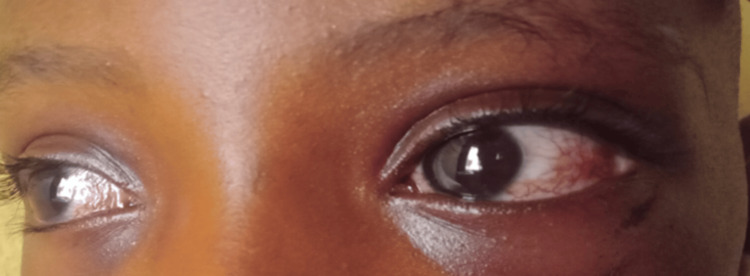
Bilateral ocular telangiectasia

Examination revealed bilateral ocular telangiectasia (Figure 1), nystagmus, and drooling of saliva. Cognitive functions, including memory, concentration, and language, were intact, and all cranial nerves were normal. Cerebellar signs were present, including dysdiadochokinesia and impaired finger-to-nose coordination. Muscle tone was reduced in all limbs, with brisk reflexes and muscle strength graded at 4/5 in the lower limbs. Bilateral Babinski responses were absent. Sensory modalities were preserved. The patient exhibited a motor ataxic gait.

**Figure 2:**
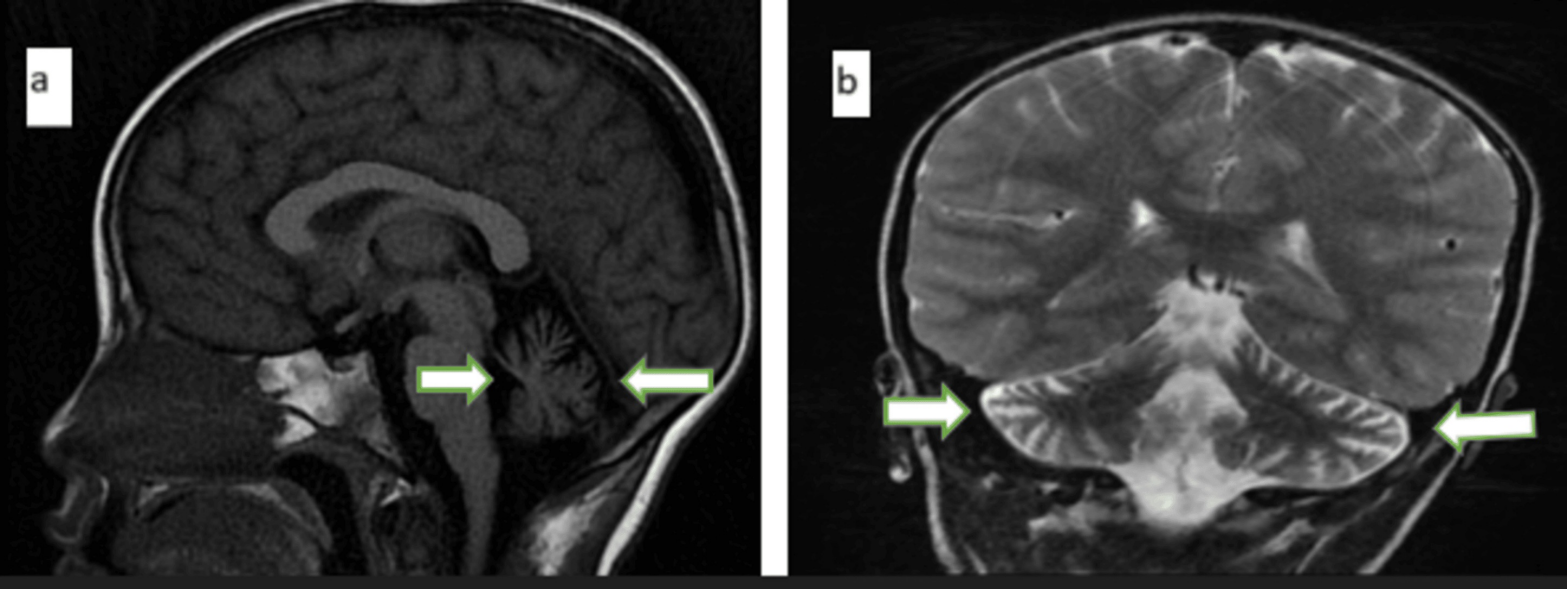
MRI of the brain (a) MRI of the brain (T1): sagittal image of the brain showing pronounced atrophy of the cerebellum. (b) MRI of the brain (T1): axial image of the brain showing pronounced atrophy of the cerebellum, most marked in the vermis.

Concerning the investigations, the complete blood count was normal, while alpha-fetoprotein was elevated at 101.04 ng/mL. Immunoglobulin (Ig) levels were within normal limits except for IgA, which was reduced to 0.24 g/L (reference range: 0.6-4.0 g/L). Brain MRI demonstrated cerebellar atrophy, most pronounced in the vermis (Figure 2). The combination of elevated AFP and MRI findings was suggestive of A-T. Confirmatory genetic testing for ATM mutations, the gold standard for diagnosis, could not be performed due to the unavailability of the testing facility.

The patient was started on physiotherapy and received the pneumococcal conjugate vaccine. Daily high-dose vitamin E (50 mg) was initiated. During an ophthalmology consultation, eyeglasses were prescribed to address visual issues associated with nystagmus. He has attended regular follow-up appointments with neurology, ophthalmology, and physiotherapy over the past 18 months. At his most recent visit, the caregiver reported that his gait had not deteriorated.

Written informed consent was obtained from the patient’s legal guardian for publication of this case report and all accompanying images.

## Discussion

A-T, also known as Louis-Bar syndrome, is a rare autosomal recessive genetic multisystemic disorder caused by a mutated ATM gene. The worldwide incidence is one in 40,000 to one in 100,000. There are very few reported cases in Nigeria [5,6]. There is no racial or sex predilection [5]. Our patient is a male, and there is no family history of A-T. The ATM gene is a phosphatidylinositol-3 kinase that phosphorylates proteins important in cell cycle checks and repairs in response to inciting stimuli such as ionizing radiation, oxidative stress, and alkylating agents. Loss of function of the ATM gene is responsible for the unchecked cell proliferation with unrepaired double-strand DNA breaks. This leads to increased susceptibility to radiosensitivity and cancer risk. The ATM gene is also important for Ig production and lymphoid cell survival. This illuminates the reason patients with an ATM mutation are at higher risk of cancer of the autoimmune and lymphatic system [6].

The clinical presentations of A-T include progressive neurological impairment, cerebellar ataxia, oculocutaneous telangiectasia, oculomotor apraxia of horizontal gaze, strabismus, X-ray hypersensitivity, immunodeficiency, and an increased susceptibility to cancer [3]. Ataxia heralds the presentation usually in the second year of life and becomes progressive with most patients requiring a wheelchair at age 10-12 years [3]. Unsteady gait was noticed in our patient at 15 months of age. The characteristic telangiectasia develops at about three years of age. This is first noticed on the bulbar conjunctivae and later on the nasal bridge, malar areas, ears, popliteal, and antecubital fossae. Ocular telangiectasia was noticed at two years of age in our patient.

About half of patients with A-T have recurrent sinopulmonary infections with varying severity depending on the level of immunodeficiency [6]. Our patient only had a decreased IgA level; however, he had no history of recurrent infections and did not show any evidence of malignancy. Patients with A-T have elevated alpha-fetoprotein levels and cerebellar atrophy on MRI; these were demonstrated in our patients. The gold standard for diagnosis is genetic testing for ATM gene mutations; however, this was not performed as such facilities are not readily available in resource-limited settings like ours.

In making the diagnosis of A-T in our patient, we relied on a combination of clinical, biochemical, and radiological criteria, as genetic testing for ATM mutations, the gold standard, was not available at the time. The key diagnostic features included the following: (1) clinical findings: early-onset progressive cerebellar ataxia, bilateral ocular telangiectasia, and nystagmus; (2) biochemical marker: markedly elevated serum alpha-fetoprotein; (3) immunological finding: selective IgA deficiency; and (4) neuroimaging - MRI of the brain showing cerebellar atrophy, especially of the vermis. The diagnosis was therefore made based on this constellation of characteristic clinical signs, supportive laboratory abnormalities, and imaging findings, in line with established diagnostic approaches in resource-limited settings where genetic confirmation is not feasible.

Treatment is supportive, as there is no definitive management to stop the progressive degeneration. Nonetheless, other manifestations, such as immunodeficiency and recurrent sinopulmonary infections, could be effectively managed. Our patient was vaccinated with the pneumococcal conjugate vaccine, had regular physiotherapy, and high-dose vitamin E 50 mg daily. Our patient developed nystagmus, and he is attending an ophthalmology clinic and is currently using glasses.

A key limitation in this case is the absence of confirmatory genetic testing for ATM mutations, which remains the gold standard for diagnosis. This limitation reflects the reality of practicing in a resource-limited setting, where access to molecular diagnostics is often unavailable. While the criteria used to make the diagnosis are strongly suggestive of A-T, the lack of genetic confirmation means that a degree of diagnostic uncertainty remains. Additionally, as with all case reports, the findings are limited to a single patient and may not be generalizable to all individuals with A-T.

## Conclusions

A-T is a rare genetic disorder typically presenting with cerebellar ataxia and ocular telangiectasias. Its wide range of additional manifestations can make diagnosis challenging. Low immunoglobulin levels, elevated alpha-fetoprotein, and brain MRI demonstrating cerebellar atrophy are strongly suggestive of A-T. While definitive diagnosis requires genetic testing for ATM mutations, such testing is often unavailable in resource-limited settings. In this case, the diagnosis remains clinical or probable rather than definitive due to the absence of genetic confirmation.
